# Pseudo-Allergies in the Emergency Department: A Common Misdiagnosis of Hypersensitivity Type 1 Allergic Reaction

**DOI:** 10.7759/cureus.46536

**Published:** 2023-10-05

**Authors:** Akram M Eraky, Alisha Wright, Douglas McDonald

**Affiliations:** 1 Emergency Medicine, Freeman Health System, Joplin, USA; 2 Emergency Medicine, Kansas City University of Medicine and Biosciences, Kansas, USA

**Keywords:** hereditary angioedema, leukotrienes, pseudo-allergy, scombroid poisoning, bradykinin mediated angioedema, histamine, drug-induced angioedema, type 1 hypersensitivity, hypersensitivity reactions, allergy and anaphylaxis

## Abstract

Type-1 hypersensitivity reaction represents an acute IgE-mediated reaction that can cause life-threatening conditions, such as anaphylactic shock, angioedema, and airway obstruction. Other reactions that can mimic type-1 hypersensitivity reactions include IgE-independent mast cell degranulation, bradykinin-mediated reactions, leukotrienes-mediated reactions, and pseudo-allergies. We use the term pseudo-allergy in this article for histamine-mediated reactions that are mast cell-independent. We did not discuss pseudo-allergic reactions that are not acute or life-threatening, such as celiac disease, Heiner’s syndrome, eosinophilic esophagitis, and food protein-induced enterocolitis in our article because the emergency department is not the primary location to diagnose or treat these reactions. Herein, we present some allergic-like reactions that can be life-threatening, such as scombroid food poisoning (SFP), bradykinin-induced angioedema, IgE-independent angioedema, opioid-induced angioedema, and non-steroidal anti-inflammatory drug (NSAID)-induced hypersensitivity and angioedema. These reactions may have different treatments based on their mechanism of reaction. Histamine-mediated reactions, such as SFP, histamine-mediated angioedema, and mast cell degranulation induced by NSAIDs, and opioids can be treated with antihistamines, epinephrine, and corticosteroids. Bradykinin-induced angioedema, including hereditary angioedema and acquired angioedema, can be treated with fresh frozen plasma. Hereditary angioedema can be treated with many FDA-approved targeted medications, such as plasma-derived C1-INH, plasma kallikrein inhibitor (Ecallantide), and selective bradykinin-2 receptor antagonist (Icatibant). However, these targeted agents are not well-studied enough to be used for acquired angioedema. It is crucial for emergency medicine physicians to be familiar with and predict these reactions to prevent misdiagnosis, be prepared to treat these life-threatening conditions appropriately without delay and eliminate patients’ exposure to any unnecessary investigations or treatments.

## Introduction and background

Hypersensitivity reactions are excessive immune responses to antigens. They are classified into four categories according to their mechanism of action. Type I hypersensitivity reactions result from histamine release by IgE-mediated mast cell degranulation. Type II hypersensitivity reactions result from the cytotoxic effect mediated by the direct effect of IgG and IgM, while type III hypersensitivity reactions are mediated by the deposition of immune complexes in body tissues. Of interest, Type VI hypersensitivity reactions are mediated by T-cells and in contrast to the other types of hypersensitivity reactions that occur within 24 hours, type VI reaction is considered a delayed reaction that can happen 12 to 72 hours after exposure to an allergen [1-3].

**Figure 1:**
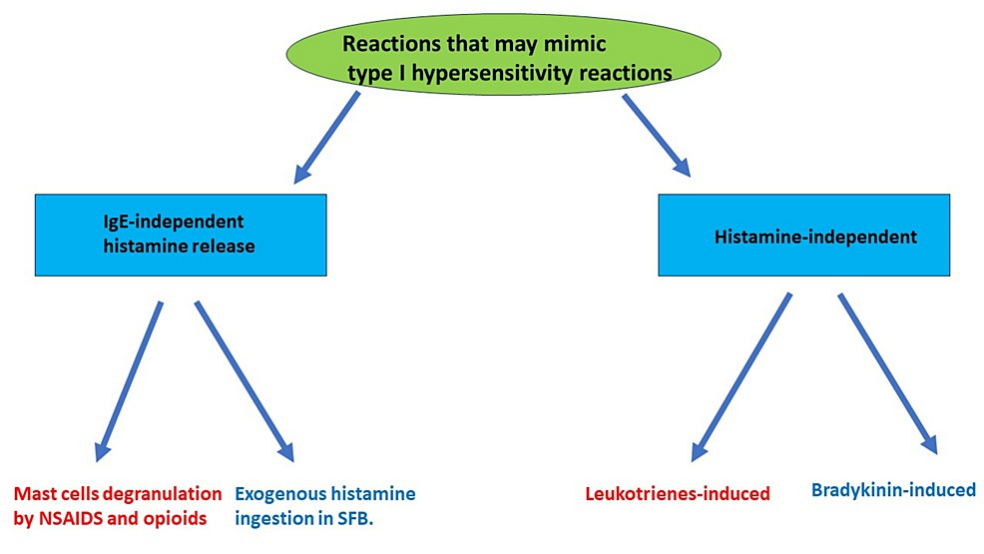
Hypersensitivity type I-like reactions that can be life threatening. Reactions that may mimic type-1 hypersensitivity reactions include (1) IgE-independent mast cell degranulation that can induced by some medications, such as NSAIDs and opioids, (2) exogenous histamine ingestion in SFB, (3) leukotrienes-induced reaction by NSAIDs, and (4) bradykinin-induced angioedema [4-7]. SFP: scombroid food poisoning. Figure credit: Akram M. Eraky

In contrast to the other three types of hypersensitivity, type I hypersensitivity reactions represent an acute IgE-mediated reaction that can cause life-threatening conditions, such as anaphylactic shock, angioedema, and airway obstruction [1-3]. Other reactions that can mimic type-1 hypersensitivity reactions include IgE-independent reactions that can be classified into two groups. The first group includes IgE-independent degranulation of mast cells that can indued by some medications, such as opioids and NSAIDs, while the second group of IgE-independent reactions includes mast cells- independent allergic reactions that result from ingestion of exogenous histamine that is produced outside the human body [1-5]. Bradykinin-mediated reactions and leukotrienes-mediated reactions are also considered reactions that can mimic type I hypersensitivity reactions and will be discussed in our review (Figure 1) [4]. We use the term pseudo-allergy in our review for histamine-mediated reactions that are mast cell-independent [4,5].

In our literature review, we found many non-IgE-mediated and pseudo-allergic reactions that are not acute or life-threatening, such as celiac disease, Heiner’s syndrome, eosinophilic esophagitis, and food protein-induced enterocolitis [4,6,7]. We did not discuss these reactions in our article because the emergency room is not the primary location to diagnose or treat these reactions and our focus in this review is to discuss the life-threatening reactions that may mimic type I hypersensitivity reaction and result in angioedema, anaphylaxis, or/and anaphylactic shock.

In this review, we present some allergic-like reactions that can be life-threatening, such as (1) SFP, (2) bradykinin-induced angioedema, (3) IgE-independent angioedema, such as opioid-induced angioedema, and (4) non-steroidal anti-inflammatory drug (NSAID)-induced hypersensitivity and angioedema. These reactions may have similarities in their clinical picture with regular allergic reactions. However, they may need different management [2-7]. It is crucial for emergency medicine physicians to be familiar with and predict these reactions to prevent misdiagnosis. Additionally, to be prepared to treat these life-threatening conditions appropriately without delay and eliminate patients’ exposure to any unnecessary testing or treatments.

## Review

Exogenous histamine ingestion

Histamine-mediated reactions that are Ig-E independent and may mimic type I hypersensitivity reactions include mast cell degranulation which will be discussed later, and mast cell-independent histamine increase in the body through exogenous histamine ingestion which will be discussed in this section.

Hypersensitivity reactions to food have been increasingly reported in the last decade. Of interest, recent studies show that food allergies affect one in four adults in the Western world [4,6,7]. Hypersensitivity reactions to food can be classified into IgE-mediated allergic reactions, non-IgE-mediated allergic reactions, and non-allergic food intolerance [4]. One of the non-allergic food intolerance reactions that can mimic a true allergic reaction is SFP [4,6]. SFP is a common hypersensitivity reaction to food throughout the world. It represents 40% of food toxicity in the United States and the European Union. In New Zealand, France, and Denmark, the incidence ranges from two to five cases per million people, while in Hawaii, the incidence is up to 31 cases per million people [5]. Many outbreaks caused by SFP throughout the world have been reported in the literature [8-11].

Some fish species, such as the Scomberesocidae and Scombridae families, contain high levels of histidine amino acid, which can be converted into histamine by decarboxylase-producing bacteria [5,12-15]. Badly stored or poorly canned fish can be contaminated by these bacteria and subsequently cause histamine-induced food hypersensitivity, which is known as SFP [5,12,13,15]. Many commonly consumed fish, such as tuna, herring, and mackerel, can cause SFP. In general, red-meat fish contain higher levels of histidine compared to white-meat fish [16].

Although this food hypersensitivity may mimic type-1 hypersensitivity reaction and can be misdiagnosed with fish allergy, it is considered a food pseudo-allergy (FPA) or false food allergy (FFA) because SFP is a mast cell-independent reaction and considered non-immunologic in origin [5,10,15,17]. Patients with SFP may present with symptoms mimicking an allergic reaction, such as flushing, itching, blurred vision, tongue edema, respiratory distress/wheezes, abdominal cramps, and diarrhea [5,9,10,13]. Treatment of SFP includes antihistamine medications. Epinephrine and corticosteroids can be used in severe cases [9,10,13,14].

In a previous report by Chen et al., these fish caused food toxicity after remaining at room temperature for only three to four hours [8]. Moreover, cooking may not decrease the risk of toxicity as histamine may not be destroyed by heat [2,4,8]. This suggests that the formation of histamine can occur in a short time; thus, keeping these fish always frozen before consumption is recommended to prevent histamine formation.

In a cross-sectional study by Zhernov et al., they found that awareness of SFP among healthcare providers is low in Tanzania. Despite SFP’s prevalence, they surprisingly found that more than half of healthcare professionals have poor knowledge about SFP [18]. This illustrates the importance of increasing awareness of this type of food poisoning among healthcare providers. It is also crucial in emergency medicine practice to differentiate between food allergy and pseudo-allergy to avoid allergy misdiagnosis and the possibility of prescribing unnecessary medications. There are two fundamental differences between SFP and true food allergies. First, re-exposure to the same non-contaminated fish meat will not induce an allergic-like reaction. Second, contaminated fish meat can cause an outbreak.

Angioedema

Type I hypersensitivity reactions may present with angioedema which is always associated with urticaria. Other reactions, that may mimic type I hypersensitivity reaction, can also present with angioedema through different mechanisms, such as IgE-independent degranulation of mast cells by opioids or NSAIDs, increased synthesis of leukotrienes by NSAIDs, and increased bradykinin production [19-23].

Angioedema is a transient swelling that can affect parts of the skin or mucosa. It lasts from a few hours up to several days. It may appear as a non-pitting, non-pruritic skin edema or swollen mucosa that can obstruct the airway [19-21]. Like urticaria, angioedema results from increased vascular permeability. In contrast to urticaria, which affects the superficial part of the dermis, angioedema affects the deeper layers of the dermis [19,20,22]. Angioedema’s association with urticaria can be used as a feature to differentiate between histamine-induced angioedema which is always associated with urticaria and bradykinin-induced angioedema which is not associated with urticaria [19-22].

Histamine-Mediated Angioedema

**Figure 2:**
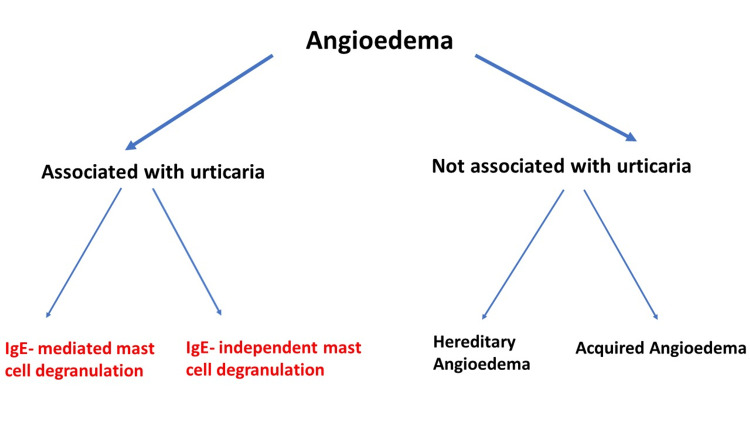
Classification of angioedema based on association with urticaria. Angioedema can be classified into two main families (histamine-mediated angioedema and bradykinin-mediated angioedema). Bradykinin-induced angioedema can be due to inherited or acquired C1-INH deficiency or dysfunction [20-23]. C1-INH: C1-esterase inhibitor. Figure credit: Akram M Eraky

Angioedema can be classified into two main families (Figure 2). The first family is known as histamine-mediated angioedema. This type of angioedema is mast cell-mediated and always associated with urticaria, which presents with a pruritic, well-circumscribed skin rash known as hives and wheals. This type responds well to antihistamines, corticosteroids, and epinephrine [20,22,23].

Histamine-mediated angioedema can be IgE-mediated and is considered a typical hypersensitivity type 1 reaction that may present with anaphylaxis, urticaria, and/or angioedema. This type can be due to a regular allergic reaction to any exogenous antigen [19,21,22]. Histamine-mediated angioedema can also be non-IgE-mediated due to the direct activation of mast cells and release of histamine without IgE interaction. Some medications, such as opioids and NSAIDs, can activate mast cell degranulation without IgE interaction and cause IgE-independent histamine-induced angioedema [20,22,23].

Opioid-Induced Angioedema

Opioids are known to cause IgE-independent mast cell degranulation and release of histamine, which can cause angioedema and allergic reactions [24-26]. Of interest, Hallberg et al. reported six cases of Tramadol-induced angioedema that caused swelling of the upper respiratory system, pharynx, or oral cavity [27]. In another case report by Masson et al., they reported a rapid development of angioedema in a patient after hydromorphone administration. This angioedema presented with swelling of the uvula, tongue, and oral mucosa and required intubation [28]. In another study by Celebioglu et al., they found that 7.3% of patients with NSAID hypersensitivity who were then treated with codeine developed hypersensitivity to codeine [29]. Although angioedema and hypersensitivity reactions to opioids are not common, they have potential adverse effects that can be life-threatening. Thus, clinicians should be aware of these reactions when using opioids.

NSAIDs-Induced Angioedema

NSAIDs can induce hypersensitivity reactions, asthma, and angioedema through two mechanisms. First, NSAIDs may cause mast cell degranulation and histamine release. Second, they can increase the production of leukotrienes by inhibiting cyclooxygenase (COX) enzymes and shifting arachidonic acid metabolism from prostaglandin formation by COX to leukotriene formation by lipoxygenase enzymes. Leukotrienes have a bronchoconstriction effect that can produce asthma-like symptoms and wheezes [30-34]. 

NSAIDs are considered the second most common cause of drug-induced hypersensitivity reactions after antibiotics [30,33]. This reaction ranges from a skin rash to anaphylactic shock. Moreover, it has a wide range of presentations, including angioedema, erythema multiforme, hypersensitivity pneumonitis, urticarial wheals, and aseptic meningitis [33,35,36]. Risk factors for developing NSAID-induced hypersensitivity include a history of asthma, chronic urticaria, and chronic rhinosinusitis. Management of NSAID-induced hypersensitivity includes drug cessation. Other treatments depend on the presentation and severity of NSAID-induced hypersensitivity, asthma, or angioedema [30,33,35].

Bradykinin-Induced Angioedema

The second family is known as bradykinin-mediated angioedema. Bradykinin is a vasodilator substance that increases vascular permeability and may cause nerve irritation. This type of angioedema is mast cell-independent and not associated with urticaria or anaphylaxis. Moreover, it does not respond to antihistamines, corticosteroids, or epinephrine [20-22,37]. Bradykinin-induced angioedema can be due to inherited or acquired C1-esterase inhibitor (C1-INH) deficiency or dysfunction. Low or dysfunctional C1-INH results in increased production of bradykinin. Acquired angioedema can also result from Angiotensin-Converting Enzyme Inhibitors (ACEI)-induced high levels of bradykinin. Hereditary angioedema is a rare autosomal dominant disorder resulting from deficiency in C1-INH (hereditary angioedema with C1 inhibitor deficiency type I) or dysfunctional C1-INH (hereditary angioedema with C1 inhibitor deficiency type II), while acquired angioedema results from consumption or inactivation of C1-INH or decrease in bradykinin degradation by ACEI [38-41]. Complement component C4 protein is the screening tool for hereditary angioedema, while C1q protein is considered a differentiating tool between hereditary versus acquired angioedema as it is low only in acquired angioedema [39-41].

ACEI, which is considered one of the most used medications worldwide, is associated with developing Angioedema. ACEI causes one-third of all angioedema-related visits to the emergency department [37-41]. ACEI is more likely to cause angioedema in patients with hereditary angioedema, smokers, users of immunosuppressive drugs, African Americans, and old people. Of interest, the prevalence of ACEI-induced angioedema is lower in diabetic patients [40,41]. ACE inhibitors reduce bradykinin and substance P breakdown and degradation by inhibiting ACE which is responsible for bradykinin degradation. As a result, there will be a bradykinin buildup that causes vasodilation and fluid extravasation, leading to edema formation in the laryngeal, gastrointestinal, upper respiratory, and oral mucosa. Patients with ACEI-induced angioedema should not be re-challenged by giving them ACEI because ACEI-induced angioedema has a high rate of recurrence [21,22,37-41].

Treatment of Bradykinin-Induced Angioedema

As we discussed earlier, antihistamines, corticosteroids, and epinephrine do not show a significant effect on bradykinin-induced angioedema as they result from increased bradykinin. The most commonly available agent for the acute management of bradykinin-induced angioedema in the emergency room is fresh frozen plasma (FFP), as it contains a small amount of C1-esterase inhibitor [20,38]. However, it may cause volume overload and allergic reactions, subsequently worsening angioedema presentation [20]. Moreover, there are many FDA-approved targeted medications for hereditary angioedema, such as a plasma-derived C1-INH, a plasma kallikrein inhibitor (Ecallantide), and a selective bradykinin-2 receptor antagonist (Icatibant). Both Icatibant and Ecallantide are administered subcutaneously, while plasma-derived C1-INH is given subcutaneously or intravenously. Theoretically, these agents may be effective with other types of bradykinin-induced angioedema, such as ACE inhibitor-induced and acquired angioedema. However, these targeted agents are not well-studied enough to be used for these types [20,22,40-42].

Many research studies supported using Icatibant to treat patients with ACEI-induced angioedema. The first randomized controlled clinical trial (RCT) discussed the efficacy of icatibant in patients with ACEI-induced angioedema was a phase 2 clinical trial conducted in Germany. In this clinical trial, Bas et al. found that the time to complete resolution is shorter in patients who were treated with Icatibant compared to patients treated with antihistaminic drugs and glucocorticoids [43]. In a previous literature review, they recommended off-label use of FDA-approved targeted medications for hereditary angioedema to treat ACEI-induced angioedema [42-45]. In many case reports, Icatibant was found to have a potential role in treating ACEI-induced angioedema [46,47]. Volans et al. found that Icatibant had an effective role in preventing intubation and reversing airway compromise in two cases after 20 minutes of injection [46]. In a retrospective study by Bas et al., they found that Icatibant may play a potential treatment in treating patients with ACEI-induced angioedema [48]. In contrast, recent two clinical trials and a meta-analysis showed no effect of Icatibant compared to a placebo [49,50]. The second RCT discussed the efficacy of icatibant in patients with ACEI-induced angioedema was a clinical trial conducted in the United States. Straka et al. found that there is no significant difference between Icatibant and placebo in treating patients with ACEI-induced angioedema [49]. The third RCT discussed the efficacy of icatibant in patients with ACEI-induced angioedema and was a phase 3 clinical trial conducted in the US, UK, Israel, and Canada. Sinert et al. found that there is no significant difference in the efficacy of Icatibant compared to placebo [50]. Moreover, a recent meta-analysis showed that there is no benefit of Icatibant over placebo. This demonstrates that the only current treatment for acquired angioedema is supportive care and there is no targeted medication for it [42] All studies that discussed the efficacy of Icatibant in our literature review are summarized below (Table 1). This highlights the importance of continuing research efforts and clinical trials to assess the effect of targeted medications in treating patients with acquired angioedema to decrease the time to recovery and prevent severe complications of ACEI.

**Table 1:** Summarization of clinical studies that discussed the efficacy of Icatibant in our literature review

Study	Type of study	Is Icatibant effective in treating ACEI-induced angioedema?
Bas et al., (2015) [43]	Clinical trial	yes
Culley et al., (2015) [44]	Literature review	Yes
Volans et al., (2013) [46]	Case report	Yes
Charmillon et al., (2014) [47]	Case report	Yes
Bas et al., (2010) [48]	Case report	Yes
Straka et al., (2016) [49]	Clinical trial	No
Sinert et al., (2017) [50]	Clinical trial	No
Jeon et al., (2019) [42]	Meta-analysis	No

Of interest, many recent clinical trials are assessing the use of Icatibant in treating other diseases. In a phase 2 clinical trial conducted in Barcelona by Malchair et al., they found that Icatibant improved mortality and pneumonia in patients with COVID-19 when a three-day Icatibant was added to the standard care of COVID-19 [51]. In another RCT by Gamboa et al., they found that continuous infusion of icatibant during hemodialysis prevented blood pressure decrease in patients complaining of intradialytic hypotension compared to placebo. Interestingly, icatibant did not have a significant effect on blood pressure in patients without intradialytic hypotension [52]. This highlights the role of vasodilators, including bradykinin, in developing intradialytic hypotension and the potential role of Icatibant as a possible prophylactic treatment in patients with intradialytic hypotension [52]. More clinical trials are encouraged to investigate the role of Icatibant in treating many pathologies including acquired angioedema.

## Conclusions

Hypersensitivity type 1 manifestations, such as anaphylactic shock, urticaria, and angioedema can be present in other reactions that have different mechanisms of action. In this review, we focus on the life-threatening reactions that may mimic type 1 hypersensitivity reactions and can be misdiagnosed in the emergency room. These reactions include IgE-independent mast cell degranulation induced by NSAIDs and opioids, bradykinin-mediated reactions, leukotrienes-mediated reactions induced by NSAIDs, and mast cells-independent allergic reactions that result from ingestion of exogenous histamine. It is important for emergency medicine physicians to be familiar with these reactions to decrease the mortality and morbidity rates in patients who develop these reactions by giving them the appropriate treatments without delay and avoiding misdiagnosis and unnecessary treatments.
